# Utilizing novel *Escherichia coli*‐specific conserved signature proteins for enhanced monitoring of recreational water quality

**DOI:** 10.1002/mbo3.1410

**Published:** 2024-04-29

**Authors:** Faizan Saleem, Enze Li, Kevin L. Tran, Bashudev Rudra, Thomas A. Edge, Herb E. Schellhorn, Radhey S. Gupta

**Affiliations:** ^1^ Department of Biology McMaster University Hamilton Ontario Canada; ^2^ Department of Biochemistry and Biomedical Sciences McMaster University Hamilton Ontario Canada

**Keywords:** conserved signature proteins, *Escherichia coli*, fecal indicators, qPCR, recreational beaches

## Abstract

*Escherichia coli* serves as a proxy indicator of fecal contamination in aquatic ecosystems. However, its identification using traditional culturing methods can take up to 24 h. The application of DNA markers, such as conserved signature proteins (CSPs) genes (unique to all species/strains of a specific taxon), can form the foundation for novel polymerase chain reaction (PCR) tests that unambiguously identify and detect targeted bacterial taxa of interest. This paper reports the identification of three new highly‐conserved CSPs (genes), namely *YahL*, *YdjO*, and *YjfZ*, which are exclusive to *E. coli*/*Shigella*. Using PCR primers based on highly conserved regions within these CSPs, we have developed quantitative PCR (qPCR) assays for the evaluation of *E. coli*/*Shigella* species in water ecosystems. Both in‐silico and experimental PCR testing confirmed the absence of sequence match when tested against other bacteria, thereby confirming 100% specificity of the tested CSPs for *E. coli*/*Shigella*. The qPCR assays for each of the three CSPs provided reliable quantification for all tested enterohaemorrhagic and environmental *E. coli* strains, a requirement for water testing. For recreational water samples, CSP‐based quantification showed a high correlation (*r* > 7, *p* < 0.01) with conventional viable *E. coli* enumeration. This indicates that novel CSP‐based qPCR assays for *E. coli* can serve as robust tools for monitoring water ecosystems and other critical areas, including food monitoring.

## Graphical abstract

This paper explores the application of a novel DNA sequence type, specifically conserved signature genes encoding conserved signature proteins (CSPs), for bacterial identification. Although our focus is on the identification of *Escherichia coli*, the same principle can be extended to identify other taxa using their respective CSPs.

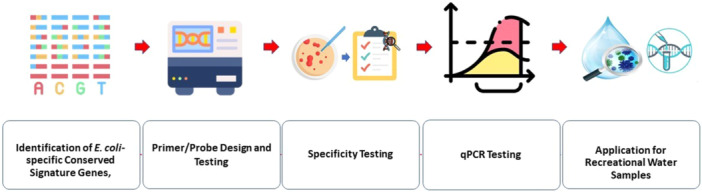

## 1 INTRODUCTION

*Escherichia coli* is a common intestinal inhabitant of homeotherms, including humans, and may be found in environmental waters due to fecal contamination (Kostyla et al., 2015). Water quality monitoring agencies commonly use *E. coli* concentration as a fecal contamination proxy indicator of freshwater quality, and *E. coli* beach action values (BAVs) are used as posting guidelines for recreational waters and protecting beachgoers from gastrointestinal illnesses (Health Canada, 2023; USEPA, 2012). Although bacteria from non‐fecal sources typically predominate in water ecosystems (Becerra‐Castro et al., 2016; Sun et al., 2019), enteric pathogens, even at low levels, can be detrimental to public health (Griffith et al., 2016; Korajkic et al., 2018). Therefore, any *E. coli* enumeration methodology should be specific only to this fecal indicator and sensitive enough to detect/quantify lower concentrations, even in the case of stochastic (outlier) contamination events.

Conventional methods of *E. coli* detection for water ecosystems usually rely on culturing‐based enumeration techniques, which have an inherent limitation of an 18–24 h delay before results are known (Dorevitch et al., 2017; Saleem et al., 2022). Furthermore, *E. coli* counts from the prior day may not be a good estimator of the following day's water quality (Saleem et al., 2023), suggesting that the changing *E. coli* concentrations within 24 h of sample collection may impact the reliability of water posting decisions. *E. coli*‐specific chromogenic media is a method of choice for culturing‐based enumeration methods, but some of these media can have high false‐positive rates due to chemical constituents in complex water samples (McLain & Williams, 2008; McLain et al., 2011). Commonly used Most Probable Number (MPN) methods, including Colilert‐18 for *E. coli* enumeration, can have >3% false positive/negative rate for freshwater *E. coli* isolates (Chao et al., 2004). Chromogenic media tests for FIB mainly rely on the activity of enzymes, including β‐galactosidase, which some environmental isolates may not express under temperature (44.5°C) required for *E. coli* testing (Alonso et al., 1998). To overcome the limitations associated with culturing‐based methods, nucleic acid amplification‐based methods have been developed for FIB like *E. coli* and *Enterococci*, including qPCR (Chern et al., 2011; Haugland et al., 2021; USEPA, 2015), Droplet Digital PCR (Cao et al., 2015; Ibekwe et al., 2020), and RNA‐based RT‐PCR (Heijnen & Medema, 2009) have been developed for water quality monitoring.

Culture‐independent molecular methods can serve as a potential alternative to conventional culturing‐based methods (Ricchi et al., 2017). In PCR‐based methods, quantification or detection of specific FIB depends on genetic markers that are taxonomically conserved only in the target and universal across all target strains. For example, *E. coli* detection PCR assays have commonly targeted hypervariable regions in universally distributed genes, including 16S rRNA gene (Clifford et al., 2012) and 23S rRNA gene (Ahmed et al., 2012). Although universal taxonomic markers have been developed for environmental water testing, inherent limitations related to the specificity and sensitivity of assays can lead to false positives/negatives (Gensberger et al., 2014; Maheux et al., 2009; Zhang et al., 2015). Additionally, universal taxonomic markers in environmental variants/isolates can have differing gene copy numbers (Kembel et al., 2012) or nucleotide polymorphisms (Hakovirta et al., 2016), which can impact the reliability and accuracy of qPCR assays for targeted taxa. Therefore, it is important to identify conserved genes that are uniquely found in a fecal indicator bacteria, based on which specific qPCR assays can be designed for environmental water testing.

In this study, we report the identification of several conserved signature proteins (CSPs) whose gene sequences are uniquely found in different *E. coli* strains. Extensive earlier work on CSPs, specific to other microbial taxa, shows that the sequences of these molecular markers provide reliable means for the demarcation of diverse microbial taxa at multiple phylogenetic depths (Gao & Gupta, 2012; Gao et al., 2009; Naushad et al., 2014). In view of their taxon‐specificity and predictive ability to be found in other members of a specific taxon, the sequences of these taxon‐specific CSPs also provide highly specific means for developing novel diagnostic tests for qualitative/quantitative assessment of specific microorganisms in biological samples, including water ecosystems (Gupta & Griffiths, 2006; Wong et al., 2014). Because of the specificity of these CSPs for a particular taxon, qPCR protocols utilizing them can overcome specificity limitations associated with other conventional universal markers, such as the 16S rRNA gene. This proof‐of‐concept study aims to identify new *E. coli/Shigella*‐specific CSPs and explore their potential use in the development of robust qPCR assays for water quality monitoring. Specific questions we address in this study are: (1) Can conserved signature proteins/genes unique to *E. coli* (and *Shigella*) be identified? (2) Can CSPs/genes be used to develop a qPCR protocol for potential water monitoring strategies? (3) Is there a good correlation between *E. coli/Shigella*‐specific CSPs gene copies and *E. coli* colony forming units (CFUs) from recreational water samples?

## 2 EXPERIMENTAL PROCEDURES

### 2.1 Identification of *E. coli* and *Shigella* spp. conserved signature proteins/DNA sequences

*E. coli* and *Shigella‐*specific Conserved Signature Proteins/DNA sequences were identified by methods used in our previous studies (Gao & Gupta, 2007; Gupta & Mathews, 2010; Gupta & Mok, 2007). Local BLASTp (Altschul et al., 1990) searches were initially conducted on individual proteins from *Escherichia coli* str. K‐12 substr. MG1655 against a database of >2000 different genomes, including>500 genomes for available *Enterobacterales* species and >200 genomes for diverse *E. coli/Shigella* strains. Based on these BLASTp searches, candidate *E. coli* signature proteins were identified for which all significant BLASTp hits were for *E. coli/Shigella* strains, and the homologs for these proteins were either not found in other bacteria or their E values were <1e‐3. Additional BLASTp searches were conducted on the protein sequences of candidate *E. coli* CSPs against the NCBI nonredundant (nr) database without the low‐complexity filter, and the top 5000 hits were examined. Based on these BLASTp searches, those proteins were identified where all significant BLASTp hits (E value < 1e‐3) were for *E. coli/Shigella* strains, and the protein was broadly found in >1000 *E. coli*/*Shigella* strains (Gao & Gupta, 2007; Gupta & Mathews, 2010; Gupta & Mok, 2007). The genes for three of the proteins identified by these searches (*YahL*, *YdjO*, and *YjfZ*) were chosen for these studies.

### 2.2 Primer/probe design and in‐silico specificity testing

For the qPCR assays, PCR primer sets were designed for the three *E. coli*/*Shigella* CSPs to be less than 120 bp in size for efficient PCR amplification. The sequences of PCR primers and qPCR probes for the three CSPs are indicated in Table 1. Specifically, the primers for *YahL*, *YdjO*, and *YjfZ* qPCR assays generated amplicon sizes of 112, 98, and 114 bases, respectively. The in‐silico specificity of these primers was tested using Primer‐BLAST (Ye et al., 2012) against NCBI nr and RefSeq genome databases with default parameters and specifying organism type as ‘bacteria.’ Additionally, in‐silico amplification specificity was also tested at the phylum level by performing separate searches against each bacterial phylum in the RefSeq genomes database. Probes were designed using specific quality criteria (Lim et al., 2011): (1) Location of the probes was kept in close proximity to one of the primers, (2) Melting temperature of the probes was kept at 5°–10° higher than the primers, and (3) GC content was kept between 35% and 65%. Probes were aligned against the NCBI RefSeq databases to check for specificity.

**Table 1:** Primers and probes targeting *E. coli/Shigella*‐specific CSPs for qPCR assays.

Gene	Primer/probe	Sequence (5′–3′)	Amplicon size (bp)
*YahL*	Forward	ACAGACGCGCCCATTAAGC	112
	Reverse	CGTCCAGAACAGAGAGCAATAA	
	Probe	(FAM*)‐AGGCGCTTGCGCAT GGATTATT‐(MGBNFQ*)	
*YdjO*	Forward	TTCTCGCTACAGGCACATTC	98
	Reverse	GGCGATGCATACTGACTCAT	
	Probe	(FAM*)‐TGAGCCAGGAATGTATTG ATAAGTTGGACA‐(MGBNFQ*)	
*YjfZ*	Forward	CAACAGGACGTATGCTCTATCG	114
	Reverse	GCCGTAAACCTTCTGCTAACTC	
	Probe	(FAM*)‐ACCTCAGCTTTAGACGA AATATATGGTGGT‐(MGBNFQ*)	

Abbreviations: *[FAM], 6‐carboxyfluorescein (fluorophore); *[MGBNFQ]: minor groove binding and nonfluorescent quencher.

### 2.3 Bacterial strain growth and in‐vitro specificity testing

The experimental specificity of the PCR primers was examined using negative controls, including *Citrobacter rodentium (Enterobacteriaceae)* and *Serratia marcescens* (*Pseudomonadota*) as in‐group negative controls, and *Micrococcus luteus* (*Actinomycetota*), *Bacillus subtilis* (*Bacillota*), and *Staphylococcus epidermis* (*Bacillota*) as non‐*Pseudomonadota* or out‐group negative controls. Bacterial strains were grown on LB (Luria–Bertani) agar plates overnight at 37°C. DNA was extracted from single colonies by incubation at 98°C for 5 min in 30 µL of 0.2% SDS (Tris‐EDTA) lysis buffer (Packeiser et al., 2013). The DNA concentration in lysate was measured using a QUBIT Fluorometer (dsDNA High‐Sensitivity Assay kit, Thermo Fisher Scientific, USA) to ensure successful DNA extraction. Initial primer pair specificity testing was performed using cell lysate from *E. coli* K12, *C. rodentium*, *S. marcescens*, and *M. luteus*, with 16S rRNA amplification as PCR reaction positive control and non‐template control. The primers and probes (qPCR assays) for the three CSPs were tested with *E. coli* K12 as positive control, negative controls (Discussed earlier), and wastewater DNA as environmental sample positive control. To test for the PCR specificity and nucleotide identity of CSPs in microbially complex samples (wastewater samples), larger amplicon fragments (600–700 bp) for each CSP (Appendix Table A1) were amplified in a total of 25 µL PCR reaction mix containing 1 µL of each primer (10 µm), 12.5 µL of Environmental master mix 2.0 (Thermo Scientific USA), and 10.5 µL of nuclease‐free water and 1 µL of DNA extracted from wastewater samples. PCR amplification cycle consisted of initial denaturation at 98°C for 10 min, followed by 35 cycles of 98°C for 30 s, annealing at 60°C for 30 s, and extension at 72°C for 60 s, followed by final extension at 72°C for 5 min. Amplified PCR fragments for each CSP were then purified using Monarch DNA Gel Extraction Kit (New England Biolabs), followed by Sanger sequencing on the SeqStudio Flex Genetic Analyzer at Farncombe Sequencing Institute (McMaster University). Sequenced CSP fragments were aligned against NCBI nr and RefSeq genome databases to validate that the amplified fragments correspond to *E. coli/Shigella*.

### 2.4 qPCR assay development and sensitivity testing

PCR fragments (98–114 bp) for each assay were purified using Monarch DNA Gel Extraction Kit (New England Biolabs), followed by DNA quantification and copy number calculations. To avoid inherent *E. coli* DNA contamination from master mixes (Palomino‐kobayashi et al., 2022), Environmental master mix 2.0 (Thermo Scientific, USA), which contains ultra‐purified Taq Polymerase was used (https://assets.thermofisher.com/TFS-Assets/LSG/manuals/cms_079133.pdf). Purified DNA was diluted 10‐fold to generate DNA standards ranging from 10^7^ to 10^1^ gene copies/µL. Standard curves for each assay were generated in 25 µL total qPCR reaction containing 1 µL of each primer (10 µm), 1 µL of probe (100 µm), 12.5 µL of Environmental master mix 2.0 (Thermo Scientific USA), and 9.5 µL of nuclease‐free water. qPCR program included initial denaturation at 98°C for 10 min, followed by 40 cycles of 98°C for 30 s and 60°C for 30 s. Standard curves were only accepted if the coefficient of determination (R^2^) was higher than 0.95 and amplification efficiency was >90%. qPCR assay sensitivity was determined by analyzing DNA extracted from ten enterohemorrhagic *E. coli* strains (Karmali et al., 2003; Riley et al., 1983), including O98:H25‐EC3, O84:NM‐EC2, O172:NM‐EC6, O103:H25‐N00, O121:NM‐N99, O113:H21‐CL3, O5:NM‐N00, O111:NM, O121:H19, O157:H7, and seven environmental *E. coli* isolates from aquatic ecosystems (obtained from Environment Canada). Gene copies/ng of DNA were calculated using slope and intercept values from standard curves generated for each assay. Each CSP gene target exists as a single gene copy per genome, and single gene copy targets are an equivalent measure of the number of microorganisms (Harwood et al., 2014). Therefore, the lower limit of detection (LLOD) for each assay was calculated as the lower limit of quantification (LLOQ), defined as the minimum number of gene copies that can be reliably detected per reaction (Klymus et al., 2020). The lower limit of quantification (LLOQ) was calculated by analyzing dilutions of standards in the range of 2 to 10 gene copies/reaction, and the coefficient of variation between the replicates of each qPCR assay was less than 15%

### 2.5 Recreational water sample collection and *E. coli* enumeration by culture

Water sample collection from recreational beaches and *E. coli* enumerations were performed as described earlier (Saleem et al., 2022; Saleem et al., 2023). In brief, 309 water samples were collected from two freshwater beaches (Marie Curtis Park East and Sunnyside beaches) and their adjacent river mouths (Etobicoke Creek and Humber River) between May 31, 2022 and August 26, 2022. Water samples were delivered to the lab within 1 h of sample collection and processed for *E. coli* enumeration by filtering 100 mL of water sample through a 0.45 µm polycarbonate membrane filter (Millipore Corp., Bedford, MA) and incubating filters on differential coliform agar (OxoidTM) for 24 h at 44.5°C. Only the samples exceeding the USEPA *E. coli* enumeration beach action value (≥235 CFUs/100 mL, *n* = 30) (USEPA, 2012) were used for qPCR testing.

### 2.6 DNA extraction, application of CSP qPCR assays for recreational waters, and data analysis

Approximately 100 mL of water sample was filtered through a 0.22 µm nitrocellulose membrane filter (Millipore Corp.), followed by DNA extraction using the Norgen Soil Plus DNA Extraction kit (Norgen Biotek Corp., Canada), as described previously (Saleem et al., 2024). The final eluate volume of DNA was 50 µL. The DNA concentration was measured using the QUBIT fluorometer (Thermo Scientific). qPCR assays and gene copy estimation for DNA from water samples were performed as described in an earlier section. For correlation analysis, data was log‐transformed, and Shapiro‐Wilk's normality testing (Stats v3.6.2 R package) was used to determine the normal distribution, followed by either Spearman's or Pearson's methods for correlation analysis.

## 3 RESULTS

### 3.1 *E. coli*/*Shigella*‐specific conserved signature proteins/genes

Conserved signature proteins/DNA proteins/DNA sequences (CSPs) specific to *E. coli* and *Shigella* spp. were identified as described in the Methods section. Based on these studies, the genes for three CSPs (*YahL*, *YdjO*, and *YjfZ*) found uniquely in *E. coli* and *Shigella* spp. were chosen for the present work. The sequences for these three CSPs matched only *E. coli* and *Shigella* spp. when aligned against NCBI(nr/nt) and RefSeq Genomes Databases. Some characteristics of these CSPs are indicated in Table 2. Of these three CSPs, two (*YdjO* and *YjfZ*) are annotated as hypothetical/uncharacterized proteins as their cellular functions are yet to be determined.

**Table 2:** Conserved signature proteins/genes specific for *E. coli* and *Shigella* spp.

Protein name (gene symbol)	Gene ID (NCBI)	Protein length (aa)	Gene length (bp)
Uncharacterized protein (*YahL*)	944970	271	816
Hypothetical protein (*YdjO*)	917061	267	804
DUF2686 domain‐containing protein (*YjfZ*)	948719	264	795

### 3.2 In‐silico and experimental validation of primer/probe specificity based on the conserved signature proteins/genes

**Figure A1:**
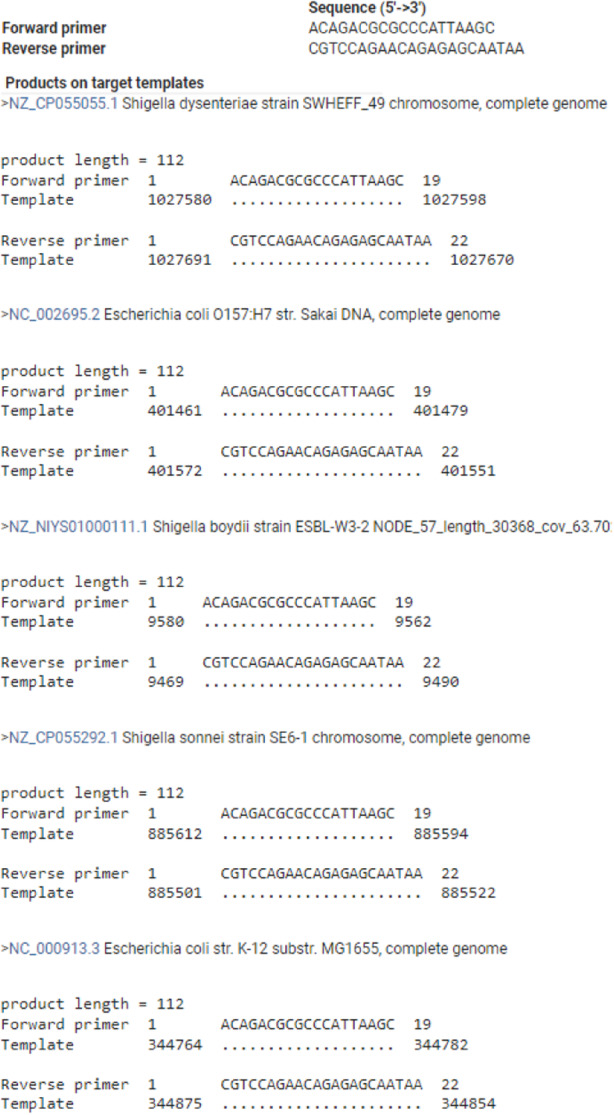
In‐silico PCR for *YaHL* primer set using NCBI RefSeq Genome Database.

**Figure A2:**
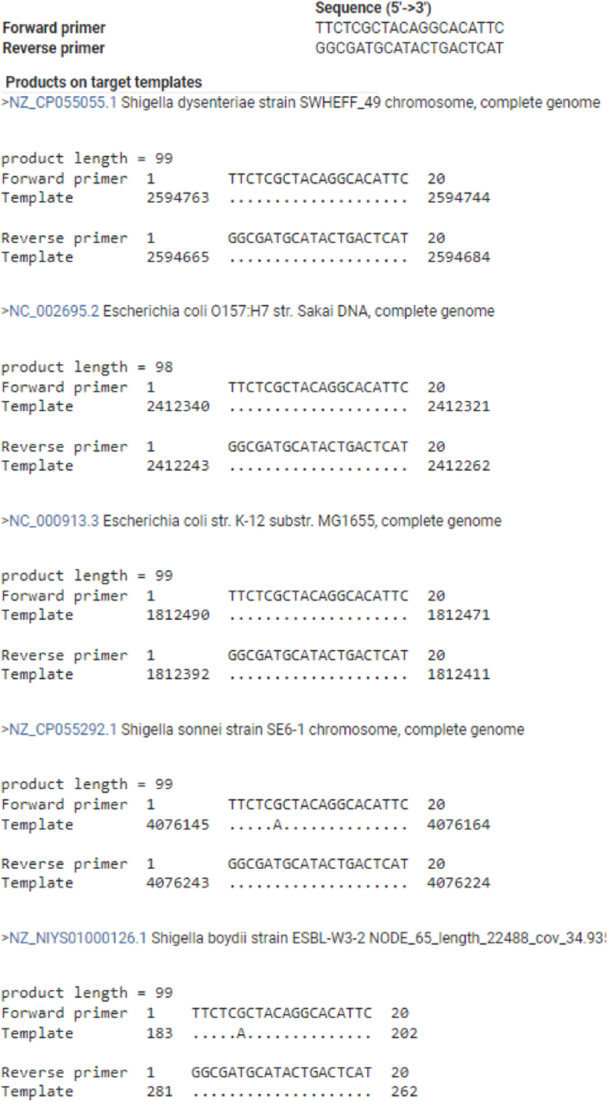
In‐silico PCR for *YdjO* primer set using NCBI RefSeq Genome database.

**Figure A3:**
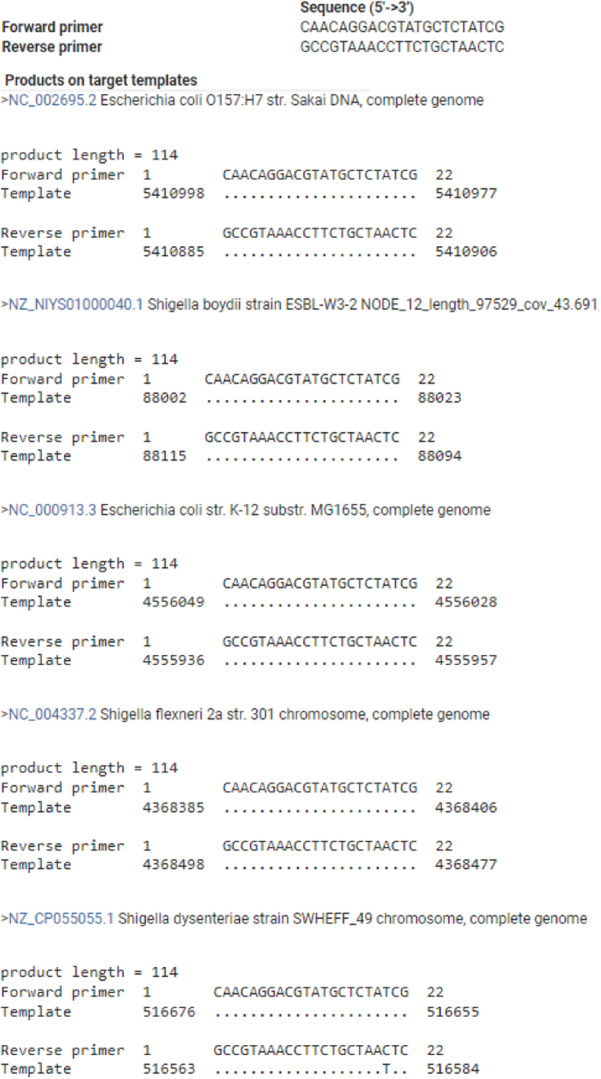
In‐silico PCR for *YjfZ* primer set using NCBI RefSeq Genome database.

**Figure A4:**
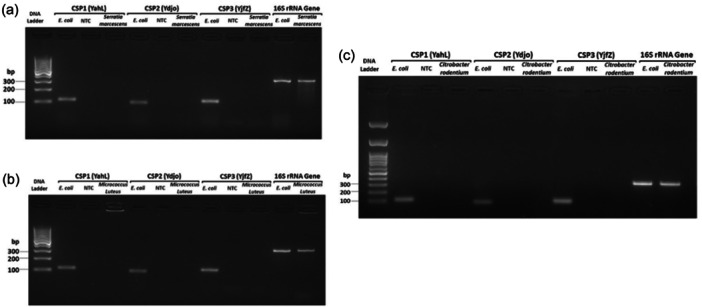
Experimental validation of *E. coli*/*Shigella*‐specific conserved signature proteins/genes based primer sets against (a) *Serratia marcescens* (in‐group negative control), (b) *Micrococcus luteus* (out‐group negative control) and (c) Citrobacter rodentium (in‐group negative control).

**Figure A5:**
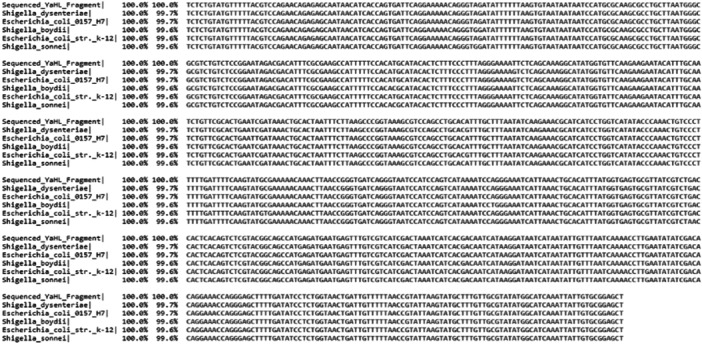
Multiple sequence alignment of sequenced YahL CSP DNA fragment (from wastewater DNA) against NCBI RefSeq reference genome database. From left to right, Lane 1: Taxonomic matches, Lane 2: Query (Sequenced Fragment) coverage, Lane 3: Percentage Identity, and Lane 4: Sequence alignment.

**Figure A6:**
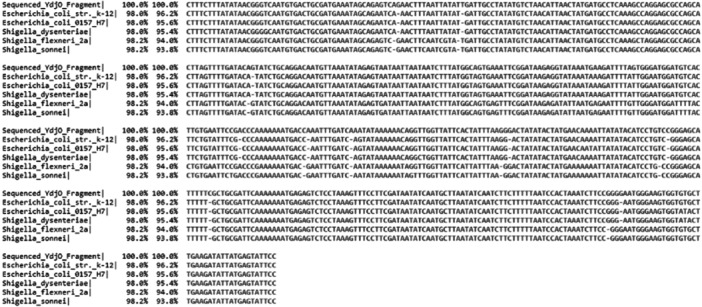
Multiple sequence alignment of sequenced *YdjO* CSP DNA fragment (from wastewater DNA) against NCBI RefSeq reference genome database. From left to right, Lane 1: Taxonomic matches, Lane 2: Query (Sequenced Fragment) coverage, Lane 3: Percentage Identity, and Lane 4: Sequence alignment.

**Figure A7:**
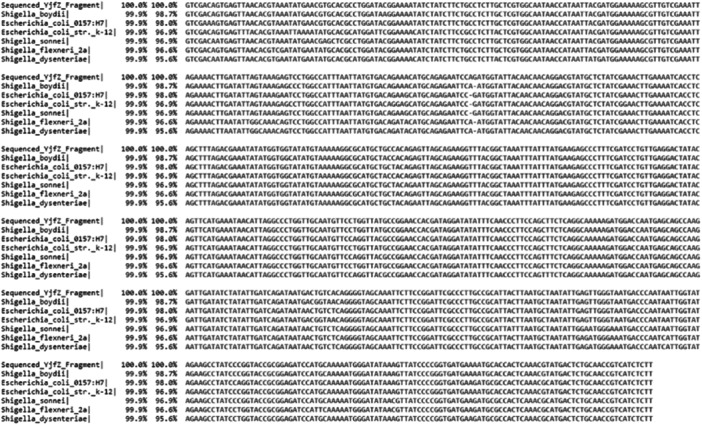
Multiple sequence alignment of sequenced YjfZ CSP DNA fragment (from wastewater DNA) against NCBI RefSeq reference genome database. From left to right, Lane 1: Taxonomic matches, Lane 2: Query (sequenced fragment) coverage, Lane 3: Percentage Identity, and Lane 4: Sequence alignment.

In‐silico PCR against NCBI nonredundant (Appendix Table A2) and RefSeq genome databases (Appendix Figures A1, A2, and A3) was used as a first step to assess the specificity of PCR amplification/detection. At the species level, in‐silico PCR hits matched with only *E. coli* for the genus *Escherichia*, while *Shigella* hits corresponded to three species (*S. dysentriae*, *S. flexneri*, and *S. sonnei*). To validate the specificity of the designed PCR primers for these three CSPs, colony PCR was performed using *E. coli* as a positive control, *C. rodentium* and *S. marcescens* as in‐group negative controls, *M. luteus* as an out‐group negative control, 16S rRNA gene as PCR reaction positive control (Appendix Figure A4). Similar to in‐silico PCR, the primer sets for all three CSPs amplified DNA fragments at the expected sizes for *E. coli*, and no amplification was observed in the examined in‐group or out‐group negative‐control species. Further, probe‐based qPCR assays for the CSPs were tested for specificity using *E. coli* DNA, wastewater samples, and negative control species (*C. rodentium*, *S. marcescens*, *M. luteus*, *S. epidermis*, and *B. subtilis*) (Appendix Table A3). Similar to the results for primer specificity tests, no amplification/fluorescence was observed for non‐target species, while the probes for all three CSPs generated positive fluorescence for *E. coli* and wastewater DNA in qPCR assays. To test the PCR specificity and nucleotide identity of CSPs from complex microbial community samples (wastewater DNA), we amplified a larger (500–700 bp) PCR fragment for each CSP, which was then sequenced (Sanger) and aligned against RefSeq reference sequences (Appendix Figures A5, A6 and A7). As expected, all three sequenced CSP fragments from wastewater DNA matched only to *E. coli* and *Shigella* species when tested against the NCBI RefSeq Genome Database. Query coverage for each CSP fragment ranged between 98% and 100%, while percentage identity was 94%–99.7%.

### 3.3 qPCR primer/probe testing and quality control analytics

qPCR assays for three CSPs were first validated on *E. coli* genomic DNA, wastewater DNA as positive controls, and negative/non‐template controls (Appendix Table A3). For three CSP assays, *E. coli* and wastewater DNA showed comparable threshold cycle (Cq) values. Following qPCR primer/probe testing, standard curves were generated for three CSP‐based qPCR assays (Table 3). The coefficients of determination for all three qPCR assays were above 0.99, and the efficiency of amplification ranged between 92% and 101%. Lower limits of quantification for *YahL*, *YdjO*, and *YjfZ* qPCR assays were determined as 2, 6, and 2 gene copies, respectively.

**Table 3:** Standard curve quality parameters for each qPCR assay from three CSPs.

Quality control parameter	*YahL*	*YdjO*	*YjfZ*
**Coefficient of determination (R** ^**2**^ **)**	0.999	0.996	0.996
**Slope**	−3.2	−3.5	−3.3
**Intercept**	40.5	38.6	38.3
**Efficiency (%)**	101	92	99
**Lower limit of quantification (LLOQ)**	2	6	2

### 3.4 Sensitivity testing using pathogenic and environmental *E. coli* strains

The sensitivity of the qPCR assays was tested against ten hemorrhagic and seven environmental *E. coli* strains (Table 4). All three qPCR assays provided positive amplification for pathogenic and nonpathogenic *E. coli* strains, with gene copies per nanogram of genomic DNA ranging between 1.5 and 5.5. Gene copies for each strain were comparatively similar between the three qPCR assays. Additionally, a significant (*p* < 0.001) positive correlation (*r*_p_ > 0.7) was observed between three qPCR assays for gene copies obtained from *E. coli* strains (Appendix Table A4). Specifically, a strong positive correlation was observed between *YahL* and *YdjO* (*r*_p_ = 0.92, *p* = 2.79E‐04), followed by *YdjO*‐*YjfZ* (*r*_p_ = 0.73, *p* = 9.40E‐04), and *YahL*‐*YjfZ* (*r*_p_ = 0.74, *p* = 9.40E‐04).

**Table 4:** Sensitivity testing of *YahL*, *YdjO*, and *YjfZ* qPCR assays for hemorrhagic and non‐hemorrhagic (environmental isolates) *E. coli* strains.

*E. coli* strain‐serotype‐seropathotype	Host	Source	Log gene copies/ng of DNA *YahL YdjO YjfZ*		
**O98:H25‐EC3‐377‐E**	Bovine	Karmali et al. (2003)	4.6	3.9	4.3
**O84:NM‐EC2‐044‐E**	Bovine	“	4.8	4.0	4.4
**O172:NM‐EC6‐484‐E**	Bovine	“	4.9	4.1	1.5
**O103:H25‐N00‐4859‐D**	Human	“	5.1	4.2	4.8
**O121:NM‐N99‐4390‐C**	Human	“	4.8	4.0	4.5
**O113:H21‐CL3‐C**	Human	“	4.9	4.2	4.6
**O5:NM‐N00‐4067‐C**	Human	“	5.3	4.6	5.2
**O111:NM‐R82F2‐B**	Human	“	5.0	4.3	4.2
**O121:H19‐CL106‐B**	Human	“	5.4	4.5	5.1
**O157:H7‐EDL933‐A**	Human	Riley et al. (1983)	5.3	4.6	5.1
**Environmental isolate**	–	Environ. Canada	4.4	3.7	4.1
**Environmental isolate**	–	“	4.3	3.9	4.0
**Environmental isolate**	–	“	5.2	4.5	4.3
**Environmental isolate**	–	“	4.4	3.9	3.7
**Environmental isolate**	–	“	4.5	4.0	3.6
**Environmental isolate**	–	“	5.1	4.5	4.6
**Environmental isolate**	–	“	4.8	4.1	4.5

### 3.5 Application of qPCR protocol for beach quality monitoring

**Figure 1:**
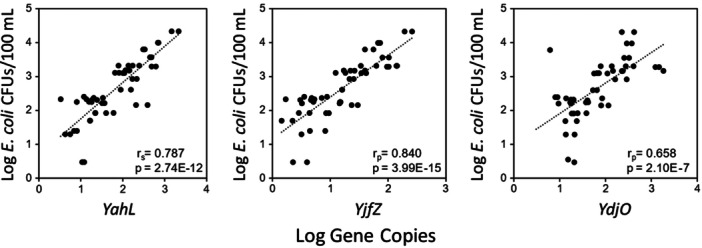
Correlation analysis between *E. coli/Shigella*‐specific conserved signature DNA sequences‐based qPCR assays and culture‐based *E. coli* colony forming units for recreational beaches and rivers.

**Figure 2:**
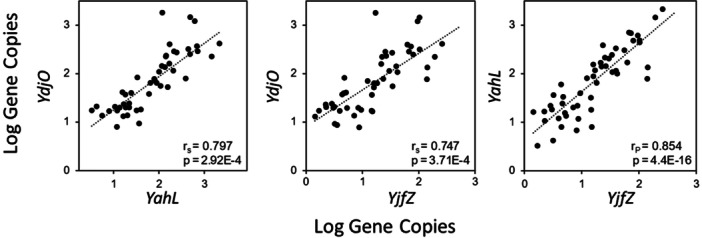
Correlation analysis between gene copies from three *E. coli/Shigella*‐specific conserved signature DNA sequences‐based qPCR assays for recreational beaches and associated rivers.

Thirty recreational water samples collected from two different beaches and associated rivers were tested for each of the *E. coli/Shigella*‐specific CSP‐based qPCR assays (probe‐based) to assess the applicability of these assays for beach monitoring applications. The detection rate of the *YahL* qPCR assay was 100% for the tested sites, followed by 96% for *YjfZ* and 93% for *YdjO* qPCR assays (Appendix Table A5). Additionally, gene copy data from three qPCR assays were compared against culturing‐based *E. coli* colony forming units (CFUs) data to assess the relationship between methods (Figure 1). Gene copies from all three qPCR assays showed a significant (*p* < 0.001) positive (*r* > 0.7) correlation with *E. coli* CFUs. Specifically, a strong correlation was observed between *YjfZ* and *E. coli* CFUs (*r*_p_ = 0.84, *p* = 3.99E‐15), followed by *YahL*‐*E. coli* CFUs (*r*_s_ = 0.78, *p* = 2.74E‐12), and *YdjO*‐*E. coli* CFUs (*r*_p_ = 0.65, *p* = 2.10E‐07). Correlation analysis was also performed to test the relationship between quantification results from the three CSP‐based qPCR assays for the same recreational waters (Figure 2). Similar to the culturable *E. coli* comparison, a strong significant (*p* < 0.001) positive correlation (*r*_p_ > 0.7) was observed for the gene copies obtained from the three different assays for the recreational water samples. Gene copies from *YahL* and *YjfZ* showed the strongest correlation (r_p_ = 0.84, *p* = 4.40E‐16), followed by *YahL*‐*YdjO* (*r*_s_ = 0.79, *p* = 2.9E‐4), and *YdjO*‐*YjfZ* (*r*_s_ = 0.74, *p* = 3.71E‐4).

## 4 DISCUSSION

Beach water quality monitoring strategies mainly rely on testing fecal indicator bacteria, including *E. coli*, using culture‐based methods, which can take up to 18–24 h and lead to delays in beach posting decisions (Dorevitch et al., 2017; Saleem et al., 2023). To date, universal taxonomic genes, including the 16S rRNA gene (Clifford et al., 2012) and the 23S rRNA gene (Ahmed et al., 2012), have been the primary targets of rapid qPCR‐based methods for *E. coli* detection. However, due to the occurrence of environmental variants of these targeted sequences, qPCR assays targeting universal taxonomic markers may lack specificity (Hakovirta et al., 2016; Maheux et al., 2009) and sensitivity (Kembel et al., 2012). Compared to conventional universal taxonomic DNA markers, Conserved Signature Proteins (CSPs)/DNA sequences represent conserved genes that are unique to specific taxonomic groups (Gao & Gupta, 2007; Gupta & Mathews, 2010; Gupta & Mok, 2007; Naushad et al., 2014). Because of their taxonomic specificity and sequence conservation, the DNA sequences of these CSPs can be targeted to detect specific taxa of interest (Gupta & Griffiths, 2006). In this proof‐of‐principle study, we identified three *E. coli/Shigella*‐specific CSPs and used them to develop a qPCR‐based protocol for testing fecal pollution in recreational freshwater beaches.

**Figure A8:**
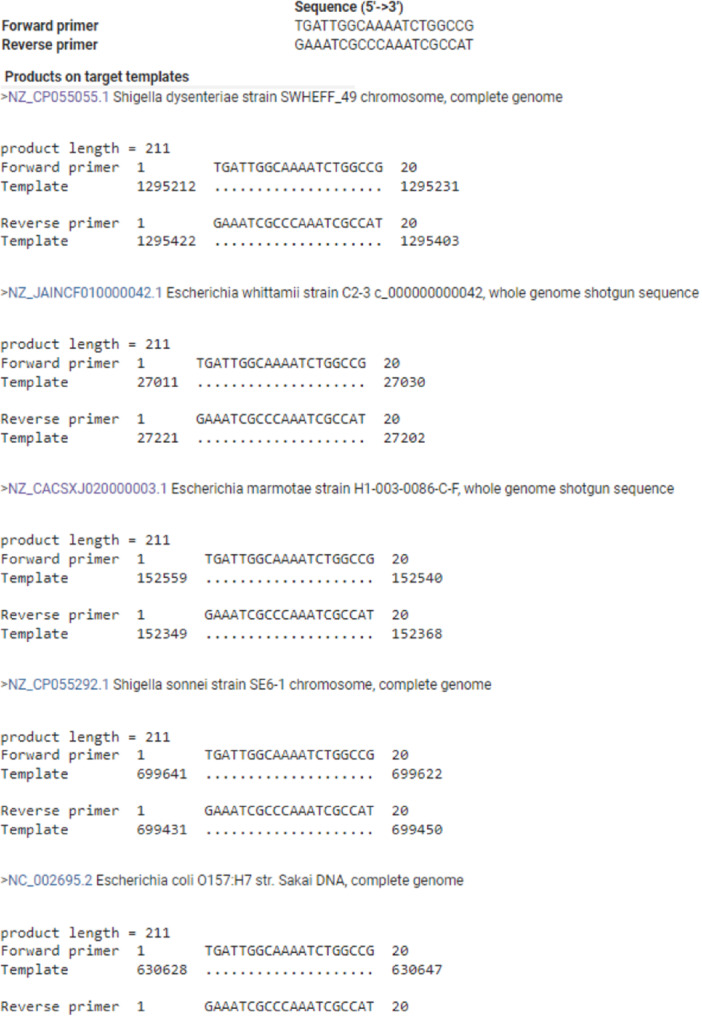
In‐silico PCR results for *ybbW* primer pair described in Walker et al. (2017) for *E. coli‐specific* qPCR assay.

**Figure A9:**
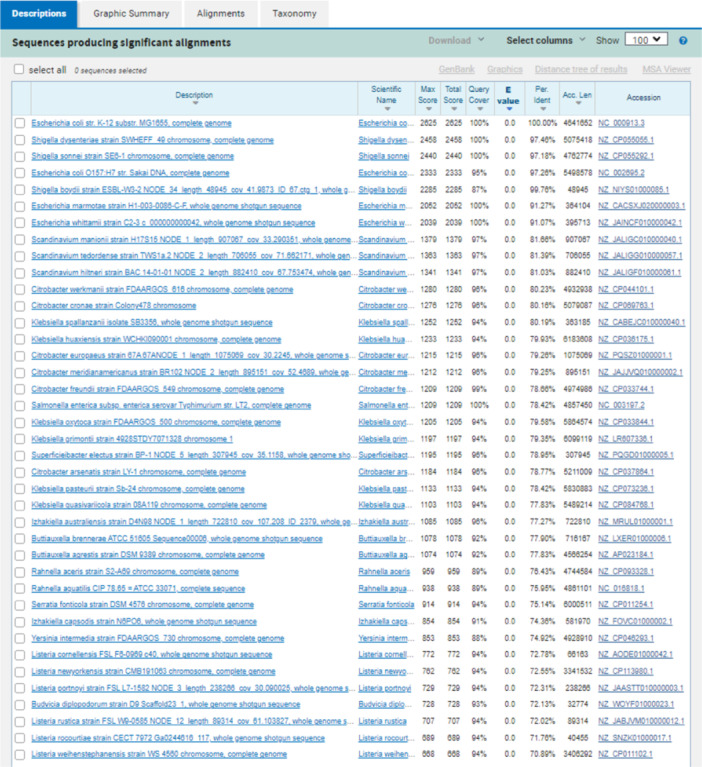
BLASTn results for the *ybbW* gene (described by Walker et al., 2017) against the NCBI RefSeq representative genomes.

In‐silico and in‐vitro primer testing validated that all three CSP sequences (*YahL*, *YdjO*, and *YjfZ*) were specific for *E. coli* and *Shigella* species, highlighting their taxonomic/evolutionary conservation among the two taxa. As *Shigella* species are phylogenetically not distinct from *E. coli* (branch in between different *E. coli* strains) (Meier‐Kolthoff et al., 2014; Sims & Kim, 2011), the shared presence of these CSPs in both *Shigella* and *E. coli* is expected. A previous study (Walker et al., 2017) developed a qPCR method targeting the *ybbW* gene, which is purportedly specific to *E. coli* and thus not present in *Shigella* or other bacteria. However, when tested (BLASTn and in‐silico PCR) against the NCBI RefSeq Representative Genome Database (Appendix Figures A8 and A9), we found *ybbW* to be also present in non‐*E. coli* species, including *Escherichia marmotae, Shigella*, and multiple non‐*Escherichia* taxa *(*>70% percentage similarity, nucleotide matched >1000 bp). Similarly, nonspecific in‐silico matches for primers and probes were observed against *Klebsiella*, *Citrobacter*, and other *Escherichia* species for the *E. coli*‐specific qPCR method based on the detection of the 23 S rRNA gene (Chern et al., 2011; Lane et al., 2020). In comparison, *YahL*, *YdjO*, and *YjfZ* were only found in *E. coli* and *Shigella* species, which signifies their value for *E. coli/Shigella* detection.

Universal taxonomic markers (including 16S and 23S rRNA genes) typically rely on a few conserved nucleotides for taxonomic characterization, but the potential for diverse single nucleotide polymorphisms within or environmental, genetic variants in conserved DNA nucleotides can lead to false positive detection of target taxa (McIlroy et al., 2011; Thorsen et al., 2016). In contrast to the other universal molecular markers used for taxonomic characterization or identification of species in environmental samples, where only a few nucleotides discriminate among different taxa, the entire coding sequences of the CSPs, which are generally quite large (in the present case ~800 bp), are specific for the members of a given taxon (*E. coli* and *Shigella* spp.). Hence, the PCR primers and qPCR probes based on these sequences provide more reliable and highly specific means for the identification/characterization of genetically diverse species such as *E. coli* in complex/ever‐evolving microbial environments such as water ecosystems.

CSP‐based qPCR assays provided positive results for all ten pathogenic (including O157:H7) and seven environmental *E. coli* isolates with comparable gene copies between tested isolates, which signifies the potential of using these assays for broad‐range environmental testing. False negative detection associated with conventional culture‐based enumeration methods is a well‐known problem (Ding et al., 2017; Kibbee & Örmeci, 2017). Specifically, *E. coli* O157:H7 can exist in a viable but not culturable (VBNC) state (Li et al., 2020; Liu et al., 2020) and cannot be detected using conventional culturing‐based water methods at 44.5°C, which can lead to underestimation of health risks. Differences in correlation strengths can be due to the inability of culturing‐based methods to culture all environmental *E. coli* isolates, including viable but not culturable cells and *E. coli* isolates, which may not grow at a specific incubation temperature (44.5°C) recommended for culturing‐based analysis (Pommepuy et al., 1996; Servais et al., 2009). Additionally, β‐glucuronidase activity‐based *E. coli* enumeration methods, including COLIFAST and COLIMINDER, can generate false positives by detecting β‐glucuronidase‐positive phenotypes belonging to *Klebsiella*, *Citrobacter*, *Aeromonas* and *Enterobacter, Yersinia and Salmonella* species (Ciebin et al., 1995; Feng & Hartman, 1982; Frampton & Restaino, 1993). if β‐d‐glucuronidase activity is either lacking (Maheux et al., 2008) or is present in lower levels (Fricker et al., 2010) in some environmental *E. coli* isolates, this could also result in underestimated quantification. In comparison, qPCR assays can also detect VBNC *E. coli* and *Shigella* species, allowing estimation of the whole spectrum of targeted taxa in complex environmental samples. However, factors including the detection of genetic material from nonviable cells (Gedalanga & Olson, 2009), environmental nucleotide variants/polymorphisms (Boyle et al., 2009; Fernández‐No et al., 2015) and some environmental strains carrying a different number of gene copies (Větrovský & Baldrian, 2013) can impact the PCR‐based quantification methods. However, a significant positive correlation between gene copy estimates of the three assays can indicate a high level of agreement between the methods.

A previously described *E. coli*‐specific RNA‐based qPCR assay (Heijnen & Medema, 2009) has limited sensitivity due to a high lower limit of quantification (LLOQ = ~10^4^ gene copies) (Walker et al., 2017). Environmental water samples can harbor diverse microbial communities with lower fecal indicator densities (Saleem et al., 2024), which may go unnoticed using qPCR assays with higher LLOQs (Walker et al., 2017). All three CSP‐based qPCR assays tested in this study demonstrated low LLOQs for recreational water samples, featuring a sensitive detection for environmental water testing. Additionally, unlike RNA‐based qPCR assays (as described previously), CSP‐based assays showed a high detection rate of *E. coli* gene copies from recreational waters, which can overcome the variable gene expression limitation associated with RNA‐based qPCR assays. Furthermore, gene copies from three CSP‐based assays showed significantly strong positive correlations with *E. coli* Colony Forming Units, indicating the potential application of CSP‐based assays as a rapid alternative to conventional culture‐based methods for beach monitoring.

In this study, we developed three independent qPCR assays using three *E. coli/Shigella*‐specific Conserved Signature Proteins/genes as targets. The potential of CSP‐based qPCR assays to detect *E. coli/Shigella* species in complex recreational water samples was also explored. All three assays can also detect *Shigella* species (*S. sonnei, S. dysenteriae, S. boydii, and S. flexneri*) of public health concern (Health Canada, 2020), which strengthens the potential of these assays. This proof of principle study demonstrates the potential of Conserved Signature Proteins/DNA sequences for designing and developing taxonomically specific quantitative/qualitative molecular assays for other water‐related and clinically important organisms. Additionally, this study can serve as the foundation for future studies to assess the relationship between CSP‐based fecal indicator estimates and Beach Action Values or water quality thresholds and the development of other CSP‐based tests for clinical, food, and water quality surveillance.

## 5 CONCLUSIONS

1. *YahL*, *YdjO*, and *YjfZ* proteins/genes were identified as conserved for *E. coli* and *Shigella sp* only, and in‐silico/in‐vitro testing validated the conservation of three CSPs, and their potential as molecular markers for developing PCR‐based assays.
2. Positive amplification was observed for Enterohemorrhagic and environmental *E. coli* strains, indicating high detection sensitivity across a range of clinical and environmental isolates of *E. coli* for CSP‐based qPCR assays.
3. *E. coli* CFUs from culturing‐based tests and CSP gene copies (qPCR) from recreational water samples showed a significant positive correlation, indicating the potential of CSP‐based qPCR assays for water monitoring applications.
4. CSP‐based qPCR assays can be a rapid testing alternative to traditional culture‐based testing methods for *E. coli* and offer a more phylogenetically targeted approach to the detection of *E. coli* and *Shigella* for water quality monitoring strategies.

## AUTHOR CONTRIBUTIONS

**Faizan Saleem**: Methodology; data curation; writing–original draft; conceptualization. **Enze Li**: Writing–review and editing. **Kevin L. Tran**: Methodology. **Bashudev Rudra**: Investigation. **Thomas A. Edge**: Writing–review and editing; conceptualization. **Herb E. Schellhorn**: Writing–review and editing; supervision; project administration; funding acquisition; conceptualization. **Radhey S. Gupta**: Conceptualization; funding acquisition; writing–review and editing.

## CONFLICT OF INTEREST STATEMENT

The authors declare no competing financial interest.

## ETHICS STATEMENT

None required.

## Data Availability

All the analytical data supporting the findings in this study is provided in the figures and tables in the article and its appendix.

## 1

**Table A1:** Primers and probes targeting *E. coli/Shigella*‐specific CSPs for conventional PCR and Sanger Sequencing.

CSP/primer name	Primer sequence (5'–3')	PCR product size (bp)
YahL‐F‐Sanger	AGCTCCGCACAATAATTTGATG	756
YahL‐R‐Sanger	CTGTCACCTAATTCCTGGACTC	
YdjO‐F‐Sanger	GCCTGGCTTTCGACTCTTT	610
YdjO‐R‐Sanger	GAATGTGCCTGTAGCGAGAA	
YjfZ‐F‐Sanger	GCCATTAAGCAATGTCCTTCAG	772
YjfZ‐R‐Sanger	AAGAGATGACGGTTGCAGAG	

**Table A2:** Summarized description of in‐silico PCR using NCBI nr database for primer sets for *YahL*, *YdjO*, and *YjfZ E. coli*/*Shigella*‐specific Conserved Signature Proteins/genes.

Conserved signature protein/genes	PCR product size (bp)	In‐silico *E. coli* matches/hits	In‐silico *Shigella* matches/hits	In‐silico non‐*E. coli* and non*‐Shigella* matches/hits	*Eschericia*/*Shigella* species matched
*YahL*	112	950	51	0	*Eschericia coli, Shigella dysenteriae, Shigella sonnei, Shigella flexneri*
*YdjO*	99	919	82	0	
*YjfZ*	114	949	57	0	

**Table A3:** Mean threshold cycle (Cq) values for *YahL, YdjO*, and *YjfZ* qPCR assays using *E. coli* DNA (positive control), Wastewater DNA (positive control), negative controls, and non‐template control.

	qPCR assay	Mean Cq Value
Wastewater DNA	*YahL*	29.2
	*YdjO*	30.0
	*YjfZ*	32.3
*E. coli* K12 Genomic DNA	*YahL*	18.8
	*YdjO*	17.2
	*YjfZ*	16.1
Non‐template/negative controls	All three qPCR assays	No amplification/fluorescence Signal

**Table A4:** Correlation between three qPCR assays using pathogenic and environmental *E. coli* isolates (*n* = 18).

Conserved signature proteins		Correlation coefficient	*p*‐value
*YahL*	*YdjO*	0.92	2.79E‐04
*YahL*	*YjfZ*	0.74	9.40E‐04
*YdjO*	*YjfZ*	0.73	9.40E‐04

**Table A5:** Detection efficiency of three qPCR assays for recreational water samples (*n* = 30).

qPCR assay	Number of positive samples	Detection efficiency (%)
*YahL*	30	100
*YdjO*	28	93
*YjfZ*	29	96

## References

[mbo31410-bib-0001] Ahmed, W. , Richardson, K. , Sidhu, J. P. , & Toze, S. (2012). *Escherichia coli* and Enterococcus spp. in rainwater tank samples: Comparison of culture‐based methods and 23S rRNA gene quantitative PCR assays. Environmental Science & Technology, 46(20), 11370–11376. 22963205 10.1021/es302222b

[mbo31410-bib-0002] Alonso, J. L. , Soriano, A. , Amoros, I. , & Ferrus, M. A. (1998). Quantitative determination of *E. coli*, and fecal coliforms in water using a chromogenic medium. Journal of Environmental Science & Health Part A, 33(6), 1229–1248.

[mbo31410-bib-0003] Altschul, S. F. , Gish, W. , Miller, W. , Myers, E. W. , & Lipman, D. J. (1990). Basic local alignment search tool. Journal of Molecular Biology, 215(3), 403–410. 2231712 10.1016/S0022-2836(05)80360-2

[mbo31410-bib-0004] Becerra‐Castro, C. , Macedo, G. , Silva, A. M. T. , Manaia, C. M. , & Nunes, O. C. (2016). Proteobacteria become predominant during regrowth after water disinfection. Science of the Total Environment, 573, 313–323. 27570199 10.1016/j.scitotenv.2016.08.054

[mbo31410-bib-0005] Boyle, B. , Dallaire, N. , & MacKay, J. (2009). Evaluation of the impact of single nucleotide polymorphisms and primer mismatches on quantitative PCR. BMC Biotechnology, 9, 75. 19715565 10.1186/1472-6750-9-75 PMC2741440

[mbo31410-bib-0006] Cao, Y. , Raith, M. R. , & Griffith, J. F. (2015). Droplet digital PCR for simultaneous quantification of general and human‐associated fecal indicators for water quality assessment. Water Research, 70, 337–349. 25543243 10.1016/j.watres.2014.12.008

[mbo31410-bib-0007] Chao, K. K. , Chao, C. C. , & Chao, W. L. (2004). Evaluation of Colilert‐18 for detection of coliforms and Eschericha coli in subtropical freshwater. Applied and Environmental Microbiology, 70(2), 1242–1244. 14766614 10.1128/AEM.70.2.1242-1244.2004 PMC348937

[mbo31410-bib-0008] Chern, E. C. , Siefring, S. , Paar, J. , Doolittle, M. , & Haugland, R. A. (2011). Comparison of quantitative PCR assays for *Escherichia coli* targeting ribosomal RNA and single copy genes. Letters in Applied Microbiology, 52(3), 298–306. 21204885 10.1111/j.1472-765X.2010.03001.x

[mbo31410-bib-0009] Ciebin, B. W. , Brodsky, M. H. , Eddington, R. , Horsnell, G. , Choney, A. , Palmateer, G. , Ley, A. , Joshi, R. , & Shears, G. (1995). Comparative evaluation of modified m‐FC and m‐TEC media for membrane filter enumeration of *Escherichia coli* in water. Applied and Environmental Microbiology, 61(11), 3940–3942. 8526507 10.1128/aem.61.11.3940-3942.1995 PMC167700

[mbo31410-bib-0010] Clifford, R. J. , Milillo, M. , Prestwood, J. , Quintero, R. , Zurawski, D. V. , Kwak, Y. I. , Waterman, P. E. , Lesho, E. P. , & Mc Gann, P. (2012). Detection of bacterial 16S rRNA and identification of four clinically important bacteria by real‐time PCR. PLoS One, 7(11), e48558. 23139793 10.1371/journal.pone.0048558 PMC3490953

[mbo31410-bib-0011] Ding, T. , Suo, Y. , Xiang, Q. , Zhao, X. , Chen, S. , Ye, X. , & Liu, D. (2017). Significance of viable but nonculturable Escherichia coli: Induction, detection, and control. Journal of Microbiology and Biotechnology. 10.4014/jmb.1609.09063 27974738

[mbo31410-bib-0012] Dorevitch, S. , Shrestha, A. , DeFlorio‐Barker, S. , Breitenbach, C. , & Heimler, I. (2017). Monitoring urban beaches with qPCR vs. culture measures of fecal indicator bacteria: Implications for public notification. Environmental Health, 16, 45. 28499453 10.1186/s12940-017-0256-y PMC5429575

[mbo31410-bib-0013] Feng, P. C. , & Hartman, P. A. (1982). Fluorogenic assays for immediate confirmation of *Escherichia coli* . Applied and Environmental Microbiology, 43(6), 1320–1329. 7049088 10.1128/aem.43.6.1320-1329.1982 PMC244235

[mbo31410-bib-0014] Fernández‐No, I. C. , Böhme, K. , Caamaño‐Antelo, S. , Barros‐Velázquez, J. , & Calo‐Mata, P. (2015). Identification of single nucleotide polymorphisms (SNPs) in the 16S rRNA gene of foodborne Bacillus spp. Food Microbiology, 46, 239–245. 25475292 10.1016/j.fm.2014.08.010

[mbo31410-bib-0015] For health professionals: Shigellosis (Shigella) . Available at: https://www.canada.ca/en/public-health/services/diseases/shigella/health-professionals.html

[mbo31410-bib-0016] Frampton, E. W. , & Restaino, L. (1993). Methods for *Escherichia coli* coli identification in food, water and clinical samples based on beta‐glucuronidase detection. Journal of Applied Bacteriology. 10.1111/j.1365-2672.1993.tb03019.x 8468256

[mbo31410-bib-0017] Fricker, C. R. , Warden, P. S. , & Eldred, B. J. (2010). Understanding the cause of false negative β‐d‐glucuronidase reactions in culture media containing fermentable carbohydrate. Letters in Applied Microbiology, 50(6), 547–551. 20374452 10.1111/j.1472-765X.2010.02834.x

[mbo31410-bib-0018] Gao, B. , & Gupta, R. S. (2007). Phylogenomic analysis of proteins that are distinctive of Archaea and its main subgroups and the origin of methanogenesis. BMC Genomics, 8(1), 86. 17394648 10.1186/1471-2164-8-86 PMC1852104

[mbo31410-bib-0019] Gao, B. , & Gupta, R. S. (2012). Phylogenetic framework and molecular signatures for the main clades of the phylum Actinobacteria. Microbiology and Molecular Biology Reviews, 76(1), 66–112. 22390973 10.1128/MMBR.05011-11 PMC3294427

[mbo31410-bib-0020] Gao, B. , Mohan, R. , & Gupta, R. S. (2009). Phylogenomics and protein signatures elucidating the evolutionary relationships among the gammaproteobacteria. International Journal of Systematic and Evolutionary Microbiology, 59(2), 234–247. 19196760 10.1099/ijs.0.002741-0

[mbo31410-bib-0021] Gedalanga, P. B. , & Olson, B. H. (2009). Development of a quantitative PCR method to differentiate between viable and non‐viable bacteria in environmental water samples. Applied Microbiology and Biotechnology, 82, 587–596. 19153730 10.1007/s00253-008-1846-y PMC7419450

[mbo31410-bib-0022] Gensberger, E. T. , Polt, M. , Konrad‐Köszler, M. , Kinner, P. , Sessitsch, A. , & Kostić, T. (2014). Evaluation of quantitative PCR combined with PMA treatment for molecular assessment of microbial water quality. Water Research, 67, 367–376. 25459225 10.1016/j.watres.2014.09.022

[mbo31410-bib-0023] Griffith, J. F. , Weisberg, S. B. , Arnold, B. F. , Cao, Y. , Schiff, K. C. , & Colford Jr. J. M. (2016). Epidemiologic evaluation of multiple alternate microbial water quality monitoring indicators at three California beaches. Water Research, 94, 371–381. 27040577 10.1016/j.watres.2016.02.036

[mbo31410-bib-0024] Gupta, R. S. , & Griffiths, E. (2006). Chlamydiae‐specific proteins and indels: novel tools for studies. Trends in Microbiology, 14(12), 527–535. 17049238 10.1016/j.tim.2006.10.002

[mbo31410-bib-0025] Gupta, R. S. , & Mok, A. (2007). Phylogenomics and signature proteins for the alpha proteobacteria and its main groups. BMC Microbiology, 7, 106. 18045498 10.1186/1471-2180-7-106 PMC2241609

[mbo31410-bib-0026] Gupta, R. S. , & D. W. Mathews (2010). Signature proteins for the major clades of Cyanobacteria. BMC Evolutionary Biology, 10(1), 24. 20100331 10.1186/1471-2148-10-24 PMC2823733

[mbo31410-bib-0027] Hakovirta, J. R. , Prezioso, S. , Hodge, D. , Pillai, S. P. , & Weigel, L. M. (2016). Identification and analysis of informative single nucleotide polymorphisms in 16S rRNA gene sequences of the Bacillus cereus group. Journal of Clinical Microbiology, 54(11), 2749–2756. 27582514 10.1128/JCM.01267-16 PMC5078553

[mbo31410-bib-0028] Harwood, V. J. , Staley, C. , Badgley, B. D. , Borges, K. , & Korajkic, A. (2014). Microbial source tracking markers for detection of fecal contamination in environmental waters: relationships between pathogens and human health outcomes. FEMS Microbiology Reviews, 38(1), 1–40. 23815638 10.1111/1574-6976.12031

[mbo31410-bib-0029] Haugland, R. , Oshima, K. , Sivaganesan, M. , Dufour, A. , Varma, M. , Siefring, S. , Nappier, S. , Schnitker, B. , & Briggs, S. (2021). Large‐scale comparison of *E. coli* levels determined by culture and a qPCR method (EPA Draft Method C) in Michigan towards the implementation of rapid, multi‐site beach testing. Journal of Microbiological Methods, 184, 106186. 33766609 10.1016/j.mimet.2021.106186 PMC8650687

[mbo31410-bib-0030] Health Canada . (2023). *Canadian recreational water quality guidelines‐Indicators of fecal contamination: Overview*. Available at: https://www.canada.ca/en/health-canada/services/publications/healthy-living/recreational-water-quality-guidelines-indicators-fecal-contamination.html

[mbo31410-bib-0031] Heijnen, L. , & Medema, G. (2009). Method for rapid detection of viable *Escherichia coli* in water using real‐time NASBA. Water Research, 43(12), 3124–3132. 19476965 10.1016/j.watres.2009.04.025

[mbo31410-bib-0032] Ibekwe, M. A. , Murinda, S. E. , Park, S. , Obayiuwana, A. , Murry, M. A. , Schwartz, G. , & Lundquist, T. (2020). Comparative use of quantitative PCR (qPCR), droplet digital PCR (ddPCR), and recombinase polymerase amplification (RPA) in the detection of Shiga toxin‐producing *E. coli* (STEC) in environmental samples. Water, 12(12), 3507.

[mbo31410-bib-0033] Karmali, M. A. , Mascarenhas, M. , Shen, S. , Ziebell, K. , Johnson, S. , Reid‐Smith, R. , Isaac‐Renton, J. , Clark, C. , Rahn, K. , & Kaper, J. B. (2003). Association of genomic O island 122 of *Escherichia coli* EDL 933 with verocytotoxin‐producing *Escherichia coli* seropathotypes that are linked to epidemic and/or serious disease. Journal of Clinical Microbiology, 41, 4930–4940. 14605120 10.1128/JCM.41.11.4930-4940.2003 PMC262514

[mbo31410-bib-0034] Kembel, S. W. , Wu, M. , Eisen, J. A. , & Green, J. L. (2012). Incorporating 16S gene copy number information improves estimates of microbial diversity and abundance. PLoS Computational Biology, 8(10), e1002743. 23133348 10.1371/journal.pcbi.1002743 PMC3486904

[mbo31410-bib-0035] Kibbee, R. J. , & Örmeci, B. (2017). Development of a sensitive and false‐positive free PMA‐qPCR viability assay to quantify VBNC *Escherichia coli* and evaluate disinfection performance in wastewater effluent. Journal of Microbiological Methods, 132, 139–147. 27932085 10.1016/j.mimet.2016.12.004

[mbo31410-bib-0036] Klymus, K. E. , Merkes, C. M. , Allison, M. J. , Goldberg, C. S. , Helbing, C. C. , Hunter, M. E. , Jackson, C. A. , Lance, R. F. , Mangan, A. M. , Monroe, E. M. , Piaggio, A. J. , Stokdyk, J. P. , Wilson, C. C. , & Richter, C. A. (2020). Reporting the limits of detection and quantification for environmental DNA assays. Environmental DNA, 2(3), 271–282.

[mbo31410-bib-0037] Korajkic, A. , McMinn, B. , & Harwood, V. (2018). Relationships between microbial indicators and pathogens in recreational water settings. International Journal of Environmental Research and Public Health, 15(12), 2842. 30551597 10.3390/ijerph15122842 PMC6313479

[mbo31410-bib-0038] Kostyla, C. , Bain, R. , Cronk, R. , & Bartram, J. (2015). Seasonal variation of fecal contamination in drinking water sources in developing countries: a systematic review. Science of the Total Environment, 514, 333–343. 25676921 10.1016/j.scitotenv.2015.01.018

[mbo31410-bib-0039] Lane, M. J. , Rediske, R. R. , McNair, J. N. , Briggs, S. , Rhodes, G. , Dreelin, E. , Sivy, T. , Flood, M. , Scull, B. , Szlag, D. , Southwell, B. , Isaacs, N. M. , & Pike, S. (2020). A comparison of *E. coli* concentration estimates quantified by the EPA and a Michigan laboratory network using EPA draft method C. Journal of Microbiological Methods, 179, 106086. 33058947 10.1016/j.mimet.2020.106086

[mbo31410-bib-0040] Li, Y. , Huang, T. Y. , Ye, C. , Chen, L. , Liang, Y. , Wang, K. , & Liu, J. (2020). Formation and control of the viable but non‐culturable state of foodborne pathogen *Escherichia coli* O157: H7. Frontiers in Microbiology, 11, 1202. 32612584 10.3389/fmicb.2020.01202 PMC7308729

[mbo31410-bib-0041] Lim, J. , Shin, S. G. , Lee, S. , & Hwang, S. (2011). Design and use of group‐specific primers and probes for real‐time quantitative PCR. Frontiers of Environmental Science & Engineering in China, 5, 28–39.

[mbo31410-bib-0042] Liu, Y. , Kumblathan, T. , Uppal, G. K. , Zhou, A. , Moe, B. , Hrudey, S. E. , & Li, X. F. (2020). A hidden risk: survival and resuscitation of *Escherichia coli* O157: H7 in the viable but nonculturable state after boiling or microwaving. Water Research, 183, 116102. 32745672 10.1016/j.watres.2020.116102

[mbo31410-bib-0043] Maheux, A. F. , Picard, F. J. , Boissinot, M. , Bissonnette, L. , Paradis, S. , & Bergeron, M. G. (2009). Analytical comparison of nine PCR primer sets designed to detect the presence of *Escherichia coli*/Shigella in water samples. Water Research, 43(12), 3019–3028. 19482328 10.1016/j.watres.2009.04.017

[mbo31410-bib-0044] Maheux, A. F. , Huppé, V. , Boissinot, M. , Picard, F. J. , Bissonnette, L. , Bernier, J. L. T. , & Bergeron, M. G. (2008). Analytical limits of four β‐glucuronidase and β‐galactosidase‐based commercial culture methods used to detect *Escherichia coli* and total coliforms. Journal of Microbiological Methods, 75(3), 506–514. 18760312 10.1016/j.mimet.2008.08.001

[mbo31410-bib-0045] McIlroy, S. J. , Tillett, D. , Petrovski, S. , & Seviour, R. J. (2011). Non‐target sites with single nucleotide insertions or deletions are frequently found in 16S rRNA sequences and can lead to false positives in fluorescence in situ hybridization (FISH). Environmental Microbiology, 13(1), 33–47. 20649647 10.1111/j.1462-2920.2010.02306.x

[mbo31410-bib-0046] McLain, J. E. T. , & Williams, C. F. (2008). Seasonal variation in accurate identification of *Escherichia coli* within a constructed wetland receiving tertiary‐treated municipal effluent. Water Research, 42(15), 4041–4048. 18674793 10.1016/j.watres.2008.06.003

[mbo31410-bib-0047] McLain, J. E. T. , Rock, C. M. , Lohse, K. , & Walworth, J. (2011). False‐positive identification of *Escherichia coli* in treated municipal wastewater and wastewater‐irrigated soils. Canadian Journal of Microbiology, 57(10), 775–784. 21936679 10.1139/w11-070

[mbo31410-bib-0048] Meier‐Kolthoff, J. P. , Hahnke, R. L. , Petersen, J. , Scheuner, C. , Michael, V. , Fiebig, A. , Rohde, C. , Rohde, M. , Fartmann, B. , Goodwin, L. A. , Chertkov, O. , Reddy, T. , Pati, A. , Ivanova, N. N. , Markowitz, V. , Kyrpides, N. C. , Woyke, T. , Göker, M. , & Klenk, H. P. (2014). Complete genome sequence of DSM 30083 T, the type strain (U5/41 T) of *Escherichia coli*, and a proposal for delineating subspecies in microbial taxonomy. Standards in Genomic Sciences, 9, 2. 25780495 10.1186/1944-3277-9-2 PMC4334874

[mbo31410-bib-0049] Naushad, H. S. , Lee, B. , & Gupta, R. S. (2014). Conserved signature indels and signature proteins as novel tools for understanding microbial phylogeny and systematics: identification of molecular signatures that are specific for the phytopathogenic genera Dickeya, Pectobacterium and Brenneria. International Journal of Systematic and Evolutionary Microbiology, 64(Pt_2), 366–383. 24505075 10.1099/ijs.0.054213-0

[mbo31410-bib-0050] Packeiser, H. , Lim, C. , Balagurunathan, B. , Wu, J. , & Zhao, H. (2013). An extremely simple and effective colony PCR procedure for bacteria, yeasts, and microalgae. Applied Biochemistry and Biotechnology, 169, 695–700. 23271627 10.1007/s12010-012-0043-8

[mbo31410-bib-0051] Palomino‐kobayashi, L. A. , Pons, M. J. , & Ruiz, J. (2022). Estimation of inherent bacterial DNA contamination in a qPCR master mix: Concerns about background DNA of reagents. Minerva Biotechnology & Biomolecular, 34(4), 180. 10.23736/S2724-542X.22.02925-X

[mbo31410-bib-0052] Pommepuy, M. , Butin, M. , Derrien, A. , Gourmelon, M. , Colwell, R. R. , & Cormier, M. (1996). Retention of enteropathogenicity by viable but nonculturable *Escherichia coli* exposed to seawater and sunlight. Applied and Environmental Microbiology, 62(12), 4621–4626. 8953732 10.1128/aem.62.12.4621-4626.1996 PMC168287

[mbo31410-bib-0053] Ricchi, M. , Bertasio, C. , Boniotti, M. B. , Vicari, N. , Russo, S. , Tilola, M. , Bellotti, M. A. , & Bertasi, B. (2017). Comparison among the quantification of bacterial pathogens by qPCR, dPCR, and cultural methods. Frontiers in Microbiology, 8, 1174. 28702010 10.3389/fmicb.2017.01174 PMC5487435

[mbo31410-bib-0054] Riley, L. W. , Remis, R. S. , Helgerson, S. D. , Mcgee, H. B. , Wells, J. G. , Davis, B. R. , Hebert, R. J. , Olcott, E. S. , Johnson, L. M. , Hargrett, N. T. , Blake, P. A. , & Cohen, M. L. (1983). Hemorrhagic colitis associated with a rare *Escherichia coli* serotype. New England Journal of Medicine, 308, 681–685. 6338386 10.1056/NEJM198303243081203

[mbo31410-bib-0055] Saleem, F. , Edge, T. A. , & Schellhorn, H. E. (2022). Validation of qPCR method for enterococci quantification at toronto beaches: Application for rapid recreational water monitoring. Journal of Great Lakes Research, 48(3), 707–716.

[mbo31410-bib-0056] Saleem, F. , Schellhorn, H. E. , Simhon, A. , & Edge, T. A. (2023). Same‐day enterococcus qPCR results of recreational water quality at two Toronto beaches provide added public health protection and reduced beach days lost. Canadian Journal of Public Health, 114, 676–687. 37069453 10.17269/s41997-023-00763-8 PMC10349029

[mbo31410-bib-0057] Saleem, F. , Li, E. , Edge, T. A. , Tran, K. L. , & Schellhorn, H. E. (2024). Identification of potential microbial risk factors associated with fecal indicator exceedances at recreational beaches. Environmental Microbiome, 19, 4. 38225663 10.1186/s40793-024-00547-8 PMC10790499

[mbo31410-bib-0058] Servais, P. , Prats, J. , Passerat, J. , & Garcia‐Armisen, T. (2009). Abundance of culturable versus viable *Escherichia coli* in freshwater. Canadian Journal of Microbiology, 55(7), 905–909. 19767865 10.1139/w09-043

[mbo31410-bib-0059] Shrestha, A. , & Dorevitch, S. (2019). Evaluation of rapid qPCR method for quantification of *E. coli* at non‐point source impacted Lake Michigan beaches. Water Research, 156, 395–403. 30933697 10.1016/j.watres.2019.03.034

[mbo31410-bib-0060] Sims, G. E. , & Kim, S. H. (2011). Whole‐genome phylogeny of *Escherichia coli*/Shigella group by feature frequency profiles (FFPs). Proceedings of the National Academy of Sciences, 108(20), 8329–8334. 10.1073/pnas.1105168108 PMC3100984 21536867

[mbo31410-bib-0061] Sun, F. , Wang, Y. , Wang, C. , Zhang, L. , Tu, K. , & Zheng, Z. (2019). Insights into the intestinal microbiota of several aquatic organisms and association with the surrounding environment. Aquaculture, 507, 196–202.

[mbo31410-bib-0062] Thorsen, J. , Brejnrod, A. , Mortensen, M. , Rasmussen, M. A. , Stokholm, J. , Al‐Soud, W. A. , Sørensen, S. , Bisgaard, H. , & Waage, J. (2016). Large‐scale benchmarking reveals false discoveries and count transformation sensitivity in 16S rRNA gene amplicon data analysis methods used in microbiome studies. Microbiome, 4, 62. 27884206 10.1186/s40168-016-0208-8 PMC5123278

[mbo31410-bib-0063] USEPA . (2012). Recreational water quality criteria (p. 42). United States (U.S.) Environmental Protection Agency (EPA). EPA‐820‐F‐12‐058. Available at https://www.epa.gov/sites/default/files/2015-10/documents/rwqc2012.pdf

[mbo31410-bib-0064] USEPA . (2015). *Method 1609.1: Enterococci in water by TaqMan® quantitative polymerase chain reaction (qPCR) with internal amplification control (IAC) assay. EPA‐820‐R‐15–099*. Available at: https://www.epa.gov/sites/default/files/2015-08/documents/method_1609-1-enterococcus-iac_2015_3.pdf.

[mbo31410-bib-0065] Větrovský, T. , & Baldrian, P. (2013). The variability of the 16S rRNA gene in bacterial genomes and its consequences for bacterial community analyses. PLoS One, 8(2), e57923. 23460914 10.1371/journal.pone.0057923 PMC3583900

[mbo31410-bib-0066] Walker, D. I. , McQuillan, J. , Taiwo, M. , Parks, R. , Stenton, C. A. , Morgan, H. , Mowlem, M. C. , & Lees, D. N. (2017). A highly specific *Escherichia coli* qPCR and its comparison with existing methods for environmental waters. Water Research, 126, 101–110. 28930669 10.1016/j.watres.2017.08.032

[mbo31410-bib-0067] Wong, S. Y. , Paschos, A. , Gupta, R. S. , & Schellhorn, H. E. (2014). Insertion/deletion‐based approach for the detection of *Escherichia coli* O157: H7 in freshwater environments. Environmental Science & Technology, 48(19), 11462–11470. 25166281 10.1021/es502794h

[mbo31410-bib-0068] Ye, J. , Coulouris, G. , Zaretskaya, I. , Cutcutache, I. , Rozen, S. , & Madden, T. L. (2012). Primer‐BLAST: A tool to design target‐specific primers for polymerase chain reaction. BMC Bioinformatics, 13, 1–11. 22708584 10.1186/1471-2105-13-134 PMC3412702

[mbo31410-bib-0069] Zhang, Y. , Hong, P. Y. , LeChevallier, M. W. , & Liu, W. T. (2015). Phenotypic and phylogenetic identification of coliform bacteria obtained using 12 coliform methods approved by the U.S. environmental protection agency. Applied and Environmental Microbiology, 81(17), 6012–6023. 26116679 10.1128/AEM.01510-15 PMC4551242

